# Risk factors of one year increment of coronary calcifications and survival in hemodialysis patients

**DOI:** 10.1186/1471-2369-11-10

**Published:** 2010-06-21

**Authors:** Giorgio Coen, Andrea Pierantozzi, Daniele Spizzichino, Daniela Sardella, Daniela Mantella, Micaela Manni, Luigi Pellegrino, Andrea Romagnoli, Roberta Pacifici, Piergiorgio Zuccaro, Salvatore DiGiulio

**Affiliations:** 1 Nephrology and Hypertension Unit, Ospedale Israelitico, Rome, Italy; 2 Department of Nephrology and Dialysis, Forlanini-San Camillo Hospital, Rome, Italy; 3 Department of Nephrology and Dialysis, San Giovanni Hospital, Rome, Italy; 4 Department of Radiology, Policlinico TorVergata, Rome, Italy; 5 Istituto Superiore di Sanità, Rome, Italy

## Abstract

**Background:**

Heart and coronary calcifications in hemodialysis patients are of very common occurrence and linked to cardiovascular events and mortality. Several studies have been published with similar results. Most of them were mainly cross-sectional and some of the prospective protocols were aimed to evaluate the results of the control of altered biochemical parameters of mineral disturbances with special regard to serum calcium, phosphate and CaxP with the use of calcium containing and calcium free phosphate chelating agents. The aim of the present study was to evaluate in hemodialysis patients classic and some non classic risk factors as predictors of calcification changes after one year and to evaluate the impact of progression on survival.

**Methods:**

81 patients on hemodialysis were studied, with a wide age range and HD vintage. Several classic parameters and some less classic risk factors were studied like fetuin-A, CRP, 25-OHD and leptin. Calcifications, as Agatston scores, were evaluated with Multislice CT basally and after 12-18 months.

**Results:**

Coronary artery calcifications were observed in 71 of 81 patients. Non parametric correlations between Agatston scores and Age, HD Age, PTH and CRP were significant. Delta increments of Agatston scores correlated also with serum calcium, CaxP, Fetuin-A, triglycerides and serum albumin. Logistic regression analysis showed Age, PTH and serum calcium as important predictors of Delta Agatston scores. LN transformation of the not normally distributed variables restricted the significant correlations to Age, BMI and CRP. Considering the Delta Agatston scores as dependent, significant predictors were Age, PTH and HDL. A strong association was found between basal calcification scores and Delta increment at one year. By logistic analysis, the one year increments in Agatston scores were found to be predictors of mortality. Diabetic and hypertensive patients have significantly higher Delta scores.

**Conclusions:**

Progression of calcification is of common occurrence, with special regard to elevated basal scores, and is predictive of survival. Higher predictive value of survival is linked to the one year increment of calcification scores. Some classic and non classic risk factors play an important role in progression. Some of them could be controlled with appropriate management with possible improvement of mortality.

## Background

Coronary calcifications are of frequent occurrence in chronic renal failure both in the conservative [1,2] and hemodialysis stages [3]. They are considered to be responsible for increased cardiac events and mortality [4], since the extent of coronary calcium deposits are associated with decreased survival [5]. Calcifications are of two types, affecting media and intima. Medial artery calcifications are known to be more frequently found in diabetes mellitus and uremia. Intimal calcifications are located in the atherosclerotic plaques and denote the presence of atherosclerotic changes of the arteries [6]. However, with the currently available non invasive means, either Electron Beam or Multislice Computerized Tomography, there is no possibility to distinguish between intimal and medial calcifications. Most of the clinical studies of uremic vascular calcifications, mainly devoted to frequency of occurrence and to risk factors enhancing calcium deposits [7-10], were based on cross-sectional data. It has been found by several authors that most of patients on hemodialysis, about 80%, are affected by coronary calcifications while only 20% or less are unaffected [3,8]. Patients showing unaffected arteries remain generally free of calcium deposits when followed for long periods of time. These patients are probably protected from calcifications thanks to inhibitory factors which have been in part identified [11,12]. Above all, age and HD age are important risk factors, together with serum phosphate, serum calcium and CaxP product. Also PTH has been considered in many studies as a risk factor of coronary calcification [13] and mortality [14-16], with special regard to cases of severe hyperparathyroidism. As previously stated, most of the studies to identify risk factors published to date are of a cross-sectional type, not statistically strong enough to show a cause-effect relationship, while only a few were prospective [17-20]. In three of them mere progression of calcification scores was evaluated [17,19,20], while in another [18] the study was conducted to evaluate the effect on progression of phosphate chelating drugs. In the present study hemodialysis patients have been evaluated by Multislice CT basally and after 12-18 months. The relationship between clinical data, basal humoral values and calcification scores, and progression of heart calcifications and mortality were evaluated in order to establish relevant risk factors and their linkage to fatal events.

## Methods

Eighty-one unselected patients with end-stage renal disease on hemodialysis were studied to evaluate the evolution of coronary and heart calcification scores. This study, due to its observational nature, has not required ethical approval. Samples were collected as part of the standard care. The patients had a mean age of 59.2 ± 10.4 years and hemodialysis vintage of 82.5 ± 99.5 months. Sex distribution was 54 M/27 F. 8 patients had diabetes mellitus and 38 patients were affected by arterial hypertension. Causes of renal failure were: chronic glomerulonephritis in 19 patients; hypertension/ischemic nephropathy in 25 patients; polycystic kidney disease in 7 patients; diabetic nephropathy in 6 patients; renal malformations in 2 patients; and unknown in 22 patients. Exclusion criteria included ethanol or drug abuse, malignancy, clinical cardiovascular disease, chronic inflammatory disease, human immunodeficiency virus infection, use of steroids, antiepileptic drugs, non-steroidal anti-inflammatory drugs, estrogens and anticoagulants. All patients were treated with hemodialysis. Hemodialysis was based on standard bicarbonate dialysis using Cuprophan or Low-Flux-PolySulfone (LF-PS) membranes, 20% with acetate-free biofiltration (AFB) using Polyacrylonitrile-.AN69 (PAN-AN69 ST), or with hemodiafiltration using Helixone (FX80) or Polycarbonate (Spiraflo SG 8 Plus). The dialysis sessions lasted 4 hours and were delivered at constant blood and dialysate flow rate values of 300 and 500 ml/min, respectively. Calcium concentration in the dialysate was 1.25, or 1.5 or 1.75 mmol/L (only 3 pts).

Most patients had been treated with relatively limited doses of calcitriol administered orally (1,5 μg/wk) or intravenously (calcitriol 4,5 μg/wk), but had discontinued this treatment at the time of the study. However, 17 patients were on IV calcitriol treatment with weekly doses ranging from 3 to 6 μg and 8 patients were treated with paricalcitol, 10-16 μg per week. Calcitriol/Paricalcitol doses were adjusted to comply with KDIGO guidelines. 81% of the patients were on phosphate binders, mainly calcium salts (calcium carbonate 1,9 ± 0,8 g/day) and sevelamer (6200 ± 2010 mg/day). In the majority of patients, regular intravenous erythropoietin treatment was underway.

Blood samples for biochemical evaluations were drawn prior to a dialysis session. Serum samples were stored at -30°C until the assays. The following assays were made: serum calcium, phosphorus, alkaline phosphatase, intact PTH, serum albumin, cholesterol, triglycerides, Fetuin-A, CRP, 25-OHD and Leptin.

Serum calcium, phosphorus, alkaline phoshatase and other biochemical parameters were measured by colorimetric methods using a Roche autoanalyzer or other standard laboratory techniques. Total calcium was corrected for serum albumin using the equation: Calcium = Ca + 0.8 (4.0 - albumin, g/dl). Normal values of these variables are reported in Table 1. Serum intact PTH was measured by an IRMA (Nichols Institute Diagnostic, San Juan Capistrano, CA). Both 1-84 and "7-84" PTH species are measured together. The normal range of values is 10 to 65 pg/ml. Fetuin-A was measured with an ELISA kit (Epitope Diagnostics Inc., San Diego). Leptin concentrations were determined with a radioimmunoassay for human leptin (Mediagnost, Reutlingen, Germany), which uses a high affinity polyclonal antibody specific for this protein. Serum 25-hydroxycholecalciferol was measured with a radioimmunoassay provided by IDS (Boldon, UK), following an extraction procedure with acetonitrile. The assay measures both 25-OHD_3 _and 25-OHD_2_.

**Table 1:** Baseline Clinical, Biochemical Data, Calcification Scores and Normal Values

Parameter		Mean	Median	SD	Interquartile range	Normal value
Number	81					

Gender (M/F)	54/27					

Age, years		58,7		10,8		

HD Age, months			45		99,5	

BMI			23,4		4	18-24*

Calcium, mg/dl		9,3		1		8,5-9,5

Phosphate, mg/dl		5,4		1,4		3,5-5,5

Albumin, g/dl		3,9		0,4		3,5-5,2

Ca, corrected, mg/dl		9,3		1,1		8,5-9,5

PTH, pg/ml			300		399,9	10-65

Cholesterol, mg/dl		149		41,2		150-210

HDL, mg/dl		43		14		35-65

Cholesterol/HDL		103,6		45,6		3,1-4,7

LDL, mg/dl		82,6		32,4		60-150

Triglicerides, mg/dl			140		142	50-180

HB, g/dl		11,7		1,2		13,5-15

Kt/V		1,3		0,3		≥1,2

Fetuin-A, g/L		0,5		0,2		0,5 - 1,5

CRP, mg/dl			1		4,2	0 - 3

Alkaline Phosphatase, U/L			149		117,3	35 - 125

25 OHD, ng/ml		19,3		9		20-40

Leptin, ng/ml			6,7		8,7	2,5-15

Therapy: Calcium carbonate or Sevelamer	81,4%					

Hypertension, yes	47,4%					

SIST, mmHg		122,4		19,2		

DIAST, mmHg		71,2		11,1		

Coronary Agatston Score t 0			481		1782,5	

Coronary Agatston Score t1			528,2		2405,9	

T0, baseline scores; T1, one year scores

* Non CKD normal values

Multislice computed tomography (MSCT) was performed basally and after 12-18 months, with a 64-channel multidetector scanner (LightSpeed VCT, General Electric Medical Systems, USA). A retrospective gating technique was used to synchronize the data reconstruction with the ECG signal. Mean heart rate during MSCT was 70 ± 8 beats/min (range 50 to 85). All scans were performed with the following parameters: detector collimation 64 × 2.5, reconstruction interval 160 mm, gantry rotation time 0.35 s, tube voltage 120 Kv, tube current 300 mAs effective, field of view (FOV) 25 cm, medium smooth convolution filter, acquisition volume 12 cm (i.e. pulmonary artery bifurcation to diaphragm), cranio-caudal scan direction. Images were obtained during a single breath-hold of approximately 5 to 7 seconds. Image reconstruction was performed using three windows at 70, 75 and 80% of the time between two consecutive R-R intervals, which corresponded to the end-diastolic phase of the cardiac cycle. The phases with minimal motion were selected and used for calculating Calcium scoring.

All MSCT data were transferred to a GE AW Workstation (software version 4.2, General Electric Medical Systems, USA) for post-processing. Calcium scoring was performed on the reconstructed image sets with commercially available software (SmartScore™ 4.0, General Electric Medical Systems, USA) [21]. Calcium was scored according to the Agatston method to quantify the amount of calcification in the coronary arteries. A focus of coronary calcium was defined as the presence of four or more contiguous pixels with more than 130 Hounsfield units. The total calcium score was calculated from the sum of the individual scores of the four major epicardial coronary arteries. Informed consent was obtained from all patients.

The statistical evaluation was carried out using a personal computer equipped with a statistical package (SPSS for windows, Chicago, Illinois, release 13.0). In addition to descriptive statistics for the selected variables, Kolmogorov-Smirnov test was employed for the continuous variables to compare the observed cumulative distribution function with the normal distribution. Non parametric (Spearman) correlations were evaluated. Agatston score was measured basally and after 12-18 months. All values were normalized to 365 days, assuming a linear trend of calcification over time. The patient's survival was followed for additional 2 years. The increment in Agatston score observed between baseline and one year was called delta. Logistic models, using tertiles of the dependent variables (coronary Agatston score), were performed to examine the relationship between the coronary calcium scores (baseline and delta) and clinical and biochemical parameters. In the logistic regression models, only those independent variables that showed to have statistically significant Spearman correlation with the dependent variable were included. Although statistically associated in bivariate analysis, some variables were excluded by logistic models because of collinearity problems. Cox regression analysis, stepwise method, has been used to investigate the association between Agatston score and mortality. The Cox regression was adjusted for age, gender, HD age, calcium and PTH. In case of OR well below unity, we used a scientific notation, reporting only the exponent (E) of base 10. A p-value < 0,05 was considered statistically significant.

## Results

Clinical characteristics, serum biochemistry and calcification scores of the patients are reported in Table 1. In case of normally distributed variables, mean and standard deviation were shown, otherwise median and interquartile range were reported. Coronary calcifications were found in 71 of 81 patients. In table 2, left side, the non parametric correlations between the basal calcification scores and the basal clinical and humoral parameters are reported. Several parameters are found to be significantly correlated to the basal scores, like Age, HD Age, PTH and CRP. On the right side of Table 2, the logistic regression's output adjusted for gender is reported, considering the tertiles of basal Agatston scores as dependent variables. The OR is referred to the high tertile compared to the low tertile as reference category. Cut-off values of Agatston score tertiles are: basal score, 157,6 and 1273; delta score, 12,2 and 239. Age (p = 0,001) and HD Age (p = 0,013) are found to be significant predictors of the basal Agatston scores. Which variables have been found to predict the value of the scores after one year? Firstly, we observed a strong association between basal calcification scores values and the delta increment after one year (p = 0,001): a low basal score is associated with a low delta, while high basal score with a high increment. Then, the answer to the question is reported in Table 3 and 4, where correlations between basal values of parameters and delta scores are reported (left sides). The logistic regressions presented in those tables have been adjusted for HD age and gender. As shown in Table 3, age (p = 0.042), calcium (p = 0.047) and PTH (p = 0.027) are significant predictors in calculating the risk of increased Agatston score after one year, while Fetuin (p = 0,027) is a protective factor. In Table 4 the results of a logistic regression of three categorized delta Agatston score as dependent variable are reported: no delta coronary calcification (reference category), those with a medium increment (delta <1000) and those with severe (delta >1000) coronary calcification increment. In the model we considered as independent variables only those that resulted as statistically associated in logistic regression of table 3. Calcium (p = 0,036) is a risk factor in moderate delta increment of Agatston score, but for severe calcification other variables are statistically significant: age (p = 0,036), PTH (p = 0,030) and, as protective factor, Fetuin-A (p = 0,023).

**Table 2:** Non parametric significant correlations and logistics regressions, adjusted for gender, of basal Agatston score

Bivariate analysis			Logistic Regression		
**Variables**	**Spearman**	**p-value**	OR*	**C.I. 95%**	**p-value**

Age	0,358	0,001	1,136	1,056 - 1,223	0,001

HD Age	0,228	0,039	1,012	1,002 - 1,021	0,013

PTH	0,263	0,017	1,002	-	0,069

CRP	0,326	0,018	1,400	-	0,106

* in logistic regression dependent variables are tertiles of basal Agatston score: high *versus *low (reference category). Pseudo R square (Cox and Snell) = 0,330

**Table 3:** Non parametric significant correlations and logistics regressions, adjusted for gender and HD age, of delta Agatston scores

Bivariate analysis			Logistic Regression		
**Variables**	**Spearman**	**p-value**	OR*	**C.I. 95%**	**p-value**

Age	0,401	0,001	1,198	1,006 - 1,426	0,042

BMI	0,390	0,001	1,375	-	0,106

Calcium	0,360	0,001	4,554	1,018 - 20,384	0,047

Ca corrected	0,362	0,001	-	-	NC

PTH	0,384	0,001	1,007	1,001 - 1,013	0,027

Fetuin-A	-0,345	0,012	5,84 E-07**	4,6 E-12** - 0,074	0,017

CRP	0,321	0,020	0,890	-	0,139

* in logistic regression dependent variables are tertiles of Delta Agatston Score: increment more than 239 *versus *increment up to 12 (reference category). Pseudo R square (Cox and Snell) = 0,505

**scientific notation

**Table 4:** Logistics regressions, adjusted for gender and HD age, of categorized delta Agatston scores

Logistic Regression				
	**Variables**	**OR**	**C.I. 95%**	**p-value**

delta increment < 1000	Age	1,044	-	0,363
	
	Calcium	3,716	1,089 - 12,678	0,036
	
	PTH	1,003	-	0,132
	
	Fetuin-A	0,015	-	0,059

delta increment > 1000	Age	1,178	1,010 - 1,373	0,036
	
	Calcium	1,550	-	0,584
	
	PTH	1,005	1,001 - 1,010	0,030
	
	Fetuin-A	0,001	4,1 E-07** - 0,336	0,023

Reference category: delta increment equal to zero

Pseudo R square (Cox and Snell) = 0,499

**scientific notation

**Figure 1:**
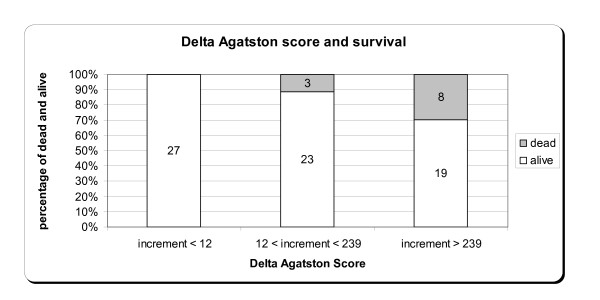
**Tertiles of delta Agatston scores and survival**.

In Figure 1, a chart showing the surviving patients, stratified by tertiles of delta Agatston after one year is shown. There were 11 death among the patients, 5 due to cardiovascular events. 72.7% of the deaths were observed among those who showed a worsening of calcifications after one year. A Cox regression, stepwise method, has been carried out to evaluate the risk factors of mortality. Age, gender, HD age, PTH, calcium and Agatston score were considered as covariates. A statistically significant cumulative mortality hazard ratio for Agatston Score (p = 0,027) was found.

We have also performed parametric analysis and used statistical models, carried out using the transformation in LN for those variables not normally distributed. The results were similar to those of non-parametric analysis (not reported in the text).

Diabetic patients have higher levels Agatston scores after one year (p < 0.042), even if they do not differ from non diabetic patients in basal conditions. The same conclusions could be drawn for arterial hypertension. Delta Agatston score was significantly higher in hypertensive (p < 0.020) compared to non hypertensive HD patients

## Discussion

In the last 10 years intense scientific interest has been dedicated to the increased cardiovascular and all-cause mortality of patients with CKD, both on predialysis and dialysis stage [4]. It is known that most of the patients with renal insufficiency, at whatever stage, die mainly due to heart disease well before reaching the stage of hemodialysis [22]. There is an accelerated atherosclerosis in uremia [23] which is partly associated to calcification of the arteries, both in the intima and in the media. Calcification of the media is prevailing in uremia, as it is with diabetic patients. Calcification of arteries, with special regard to coronary arteries can be detected by radiological techniques, which however cannot distinguish between intimal and media calcifications. However, vascular calcification is very frequent in uremia and has a recognized association with worse survival prognosis [5]. In vivo studies have shown hyperphosphatemia to be an important risk factor of death [24], and in vitro observations have shown that increased phosphate concentrations are an important factor in the calcification of the smooth muscle cells of the media, which can be able to induce differentiation of these cells in osteoblast-like cells and to induce osteogenetic changes of arterial wall and calcification [25]. Many studies have evaluated the impact of appropriate phosphate control with chelating agents on the process of vascular calcification in hemodialysis patients and compared calcium containing chelating drugs to calcium-free drugs [18,26,27], like sevelamer and lanthanum carbonate. These studies have been conducted prospectively showing that calcium-free chelating agents induce a lower increase of vascular calcification with time, compared to calcium containing chelating drugs [18]. Other studies have been planned and published to evaluate which are the major risk factors of vascular calcifications, mainly with cross-sectional protocols [9,10,28-30]. In addition to classical risk factors linked to deranged mineral metabolism, other non classical factors of vascular calcification and mortality have been identified, like Fetuin-A, Osteoprotegerin and FGF 23 [10,11,29]. Only a few studies have been conducted prospectively to evaluate progression of calcification in HD patients [17,19,20]. This study, made on a cohort of 81 hemodialysis patients, is a statistically more significant prospective analysis of the evolution of calcium deposits. The study strengthens the importance of several classical risk factors considered markers of mineral metabolism derangements, like serum calcium and PTH, and also Age, HD vintage and CRP. In addition, the study underlines the protective effect of Fetuin-A. Serum phosphate did not reach a statistically significant level, probably due to a limited range of values. In the evaluation of predictors of calcification progression, Age, serum calcium, PTH and Fetuin-A were selected as significant variables. The importance of PTH as a risk factor of vascular calcification is in apparent contrast with the histological findings of London et al [30], but in line with translational studies [31-33] and a clinical contribution [13]. In addition, the study highlights the increased mortality in the patients with higher calcification score progression and also validates earlier findings of a higher risk of progression in the patients with higher basal calcification scores [20]. Our results show that a higher increase of calcification after one year is associated to increased mortality. Monitoring progression of calcifications is of value in the survival evaluation of patients.

This study, in spite of the longitudinal approach, has some limitations mainly due to the relatively limited number of patients in the tertile groups. In addition the assumption was made that the progression of calcification deposits in the arteries was linear with time in the assessment of coronary calcium increment after one year. We used a retrospective gating in the evaluation of the score, due to frequent heart rhythm disorders in our patients, preventing the use of beta-blockers.

## Conclusions

This study of 81 hemodialysis patients examines which factors are predictive of basal coronary calcification and of their progression. Age and HD vintage, PTH and CRP are significant factors of coronary calcifications on the bivariate analysis while regression analysis selected Age and HD age. The increment of calcifications after one year was predicted by Age, serum Calcium, PTH and Fetuin-A. Categorized delta Agatston scores selected Age, serum Calcium, PTH and Fetuin-A. The delta increment of coronary calcification was predictive of mortality. As a further deduction, there are factors of progression which cannot at present be controlled, like Age and Fetuin-A, and other risk factors which could be brought into control with appropriate treatment with a potential improvement of expected survival.

## Competing interests

There are no conflicts of interest or financial and non financial competing interests for this manuscript

Giorgio Coen, MD

## Authors' contributions

GC, design of protocol, evaluation of data, draft of the manuscript. AP, evaluation of data and statistical analysis. DS, contribution to study design and statistical analysis. DS, laboratory routine and immunoassays. DM, clinical investigation and collection of data, preparation of database. MM, clinical investigation and collection of data. LP, CardioTC x-ray specialist. AR, Cardio TC, calcium scoring. RP, laboratory biochemical assays. PZ, laboratory immunoassays. SD, contribution to study design, collaboration in drafting the manuscript. All authors read and approved the final manuscript.

## Pre-publication history

The pre-publication history for this paper can be accessed here:

http://www.biomedcentral.com/1471-2369/11/10/prepub
